# Performance of Continuous Digital Monitoring of Vital Signs with a Wearable Sensor in Acute Hospital Settings

**DOI:** 10.3390/s25092644

**Published:** 2025-04-22

**Authors:** Meera Joshi, Fahad M. Iqbal, Mansour Sharabiani, Hutan Ashrafian, Sonal Arora, Kenny McAndrew, Sadia Khan, Graham Cooke, Ara Darzi

**Affiliations:** 1 Department of Surgery and Cancer, Imperial College London, London W2 1NY, UK; 2 Department of Primary Care & Public Health, School of Public Health, Imperial College London, London W6 8RP, UK; 3 Chelsea and Westminster Hospital, NHS Foundation Trust, London SW10 9NH, UK; 4 Division of Infectious Diseases, Imperial College London, London W2 1NY, UK

**Keywords:** wearable sensors, vital signs, continuous monitoring

## Abstract

Background: Continuous vital sign monitoring using wearable sensors has gained traction for the early detection of patient deterioration, particularly with the advent of virtual wards. Objective: The objective was to evaluate the reliability of a wearable sensor for monitoring heart rate (HR), respiratory rate (RR), and temperature in acutely unwell hospital patients and to identify the optimal time window for alert generation. Methods: A prospective cohort study recruited 500 patients in a single hospital. Sensor readings were compared to standard intermittent nurse observations using Bland–Altman plots to assess the limits of agreement. Results: HR demonstrated good agreement with nurse observations (intraclass correlation coefficient [ICC] = 0.66, r = 0.86, *p* < 0.001), with a mean difference of 3.63 bpm (95% LoA: −10.87 to 18.14 bpm). RR exhibited weaker agreement (ICC = 0.20, r = 0.18, *p* < 0.001), with a mean difference of −2.72 breaths per minute (95% LoA: −10.91 to 5.47 bpm). Temperature showed poor to fair agreement (ICC = 0.30, r = 0.39, *p* < 0.001), with a mean difference of −0.57 °C (95% LoA: −1.72 to 0.58 °C). A 10 min averaging window was identified as optimal, balancing data retention and real-time alerting. Conclusions: Wearable sensors demonstrate potential for reliable continuous monitoring of vital signs, supporting their future integration into real-world clinical practice for improved patient safety.

## 1. Introduction

Failure to promptly identify and respond to patient deterioration in hospital settings remains a major contributor to morbidity and mortality, as highlighted by numerous studies [1,2,3,4,5]. Acute deterioration is often preceded by changes in physiological parameters, making the measurement of vital signs a critical first step in its detection [6,7,8,9]. Subtle alterations in vital signs can occur hours before serious clinical events, such as cardiac arrest, death, or the need for intensive care unit (ICU) admission [10]. Despite this, current systems frequently fail to detect deteriorating patients in a timely manner, with approximately 39% of acute emergency admissions to critical care being referred late [4,11].

Routine vital sign monitoring includes measurements of heart rate (HR), respiratory rate (RR), oxygen saturation, blood pressure (BP), and temperature. In general wards, these observations are typically conducted intermittently, with a standard frequency of every 6–8 h [12]. The UK National Institute for Health and Clinical Excellence (NICE) recommends recording physiological observations at least every 12 h, increasing this frequency for patients showing abnormal physiology [13]. However, when deterioration is detected, monitoring frequency escalates significantly, potentially occurring every 20–30 min. This reactive approach introduces delays, potentially missing opportunities for timely intervention. Importantly, the optimal frequency for vital sign monitoring remains uncertain [14].

Digital Health Technologies, including wearable sensors, offer innovative solutions for enhancing the accuracy and timeliness of patient monitoring [15,16]. Despite their potential, formal clinical research evaluating these technologies in real-world hospital settings is limited [15]. Effective implementation requires robust collaboration between clinical researchers and industry partners, ensuring the technologies align with the needs of patients and healthcare staff [17,18]. Continuous monitoring via wearable sensors has shown promise in detecting patient deterioration earlier than standard nurse-led observations [15,19]. However, questions remain regarding their reliability, accuracy, and the ideal timing for generating alerts. Challenges such as data loss, skewed readings from outliers, and the risk of missing critical early changes when alert windows are too broad or too narrow must be addressed.

This study aims to evaluate the reliability of a wearable sensor platform for continuous monitoring of vital signs in acutely unwell patients, comparing its performance to standard nurse observations. Recognising the variability in respiratory rate measurements between static and dynamic conditions [20], this research seeks to define acceptable discrepancies between nurse and device readings, as informed by prior studies [16,21]. Additionally, the study models optimal alert timing for wearable sensors, offering insights into refining patient monitoring practices and enhancing the early detection of clinical deterioration.

## 2. Materials and Methods

### 2.1. Study Design and Setting

This prospective cohort study evaluated acutely unwell patients within 24 h of hospital admission. This study was conducted at a busy hospital in West London, UK, serving a local population of 400,000 people with 400 inpatient beds. Recruitment took place in acute medical and surgical wards between 23 January 2018 and 11 January 2019.

### 2.2. Ethics

Ethical approval for this study was obtained on 1 September 2017, from the Yorkshire & Humber—Leeds East Research Ethics Committee (reference number 17/YH/0296).

### 2.3. Recruitment and Eligibility

Patients were identified daily after consultant-led ward rounds. The acute medical and surgical teams provided a list of patients who were acutely unwell, likely to remain hospitalised for at least 24 additional hours, and capable of providing informed consent.

This study had several inclusion criteria:

1. Hospital Stay Requirement—Patients admitted to a general medical or surgical ward and expected to remain in the hospital for at least 24 h were eligible for recruitment.
2. Consultant Identification Process—Each morning, the research team approached the admitting consultants in both medicine and surgery after their ward rounds to obtain a list of eligible patients. Surgical ward rounds typically concluded around 10:00 AM, while the medical board round took place later in the day, around 12:00 PM.
3. Age Criteria—Eligible participants were aged 18 years or older, with an upper age limit of 95 years.
4. Informed Consent—All eligible patients received an information sheet outlining the sensor technology and study details. Written informed consent was mandatory before participation, ensuring that all patients understood the study requirements. The patient consent form was completed before recruitment.
5. Exclusion Criteria—Patients who were unable to provide informed consent or who later withdrew their consent were excluded from this study.

### 2.4. Sensium Vital Sign Sensor

The Sensium wearable sensor (The Surgical Company, Abingdon, UK) was used to monitor vital signs, including heart rate (HR), respiratory rate (RR), and temperature [22]. The sensor is lightweight, FDA- and CE-approved, and certified under ISO 13485 standards [23].

**Figure 1:**
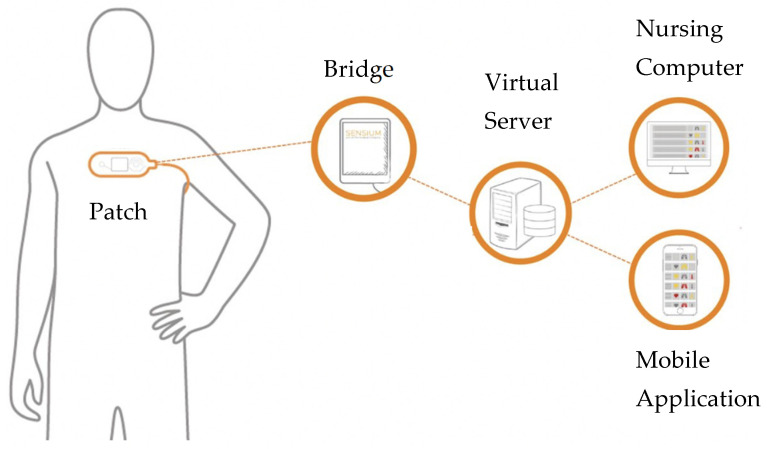
Sensor placement on a patient’s chest, placement of axillary wire, and data flow. Image reproduced with permission from Sensium (Abingdon, UK).

The sensor was either placed by trained healthcare professionals looking after the patient or the research team (Figure 1). The sensor was attached to the anterior chest wall using two standard disposable ECG electrodes (Red-Dot2560 3M, St. Paul Minn, MN, USA). Medical tape was used to ensure that the temperature probe was secured in the axilla. The axilla of the patient’s non-dominant hand was used so as not to restrict any patient movement and to limit any potential interference. A plastic strip attached to the sensor was pulled to activate the device. The sensor recorded in a sequential cyclical 2 min fashion.

### 2.5. Sensor Data Collection and Algorithm

Patented embedded algorithms processed the captured data to filter out noisy or irregular signals, minimising false alerts. The process begins with digital filtering, which removes unwanted artifacts caused by external interference or muscular electrical activity. This is followed by a decision-making stage, where the signal is evaluated against predefined rules and empirically derived thresholds to ensure accuracy. Only signals that meet these criteria are recorded and made available to the end user, ensuring a built-in quality assurance check for reliable data interpretation.

#### 2.5.1. Heart Rate (HR)

The HR algorithm, based on the Hamilton and Tompkins method [24], identifies QRS peaks in the ECG signal. Spurious peaks caused by noise are excluded, and the median HR is calculated from valid readings. Invalid signals due to excessive noise are rejected. The sensor complies with ISO 60601-2-27 standards, operating within 30–200 bpm for adults, with extended capability to detect values up to 250 bpm, though readings above 200 bpm are flagged [25].

#### 2.5.2. Respiratory Rate (RR)

The RR is derived from thoracic impedance changes. Small alternating currents passed through the ECG electrodes detect voltage variations associated with inhalation and exhalation. Peaks and troughs from the impedance waveform over a 60 s segment calculate the median RR, with invalid signals rejected [26].

#### 2.5.3. Temperature

Temperature is measured using a calibrated thermistor, a temperature-sensitive resistor placed in the patient’s axilla, providing a practical method for continuous monitoring. The temperature algorithm incorporates prior knowledge of typical core body temperature fluctuations over time to filter readings effectively. Sudden or abrupt changes, such as those caused by arm movement, are identified and excluded to minimise false alerts associated with noisy data.

### 2.6. Data Transmission

Vital sign data were processed by the sensor’s microchip and transmitted via low-power radio frequency to engineered bridges. These bridges relayed the data to a central server, enabling digital alerts to be sent to healthcare staff via smartphones and to computers on the ward [26]. Patients could wear the sensor freely on the ward, including during bathing, with data uploaded in real time. If off the ward, the sensor stored data for up to 3 h, which was then uploaded upon return.

### 2.7. Current Ward Monitoring

All patients continued to have normal bedside observations by the clinical team, and these were recorded as per standard practice on bedside paper observation charts. In the absence of specific clinical directions otherwise, bedside observations were taken at 4–6 h intervals by nurses and healthcare assistants. The reference clinical device was a conventional bedside clinical monitor (IntelliVue MP30, Philips, Amsterdam, the Netherlands). This device measures HR, blood pressure, and oxygen saturation. Tympanic temperature was taken using the Braun Thermoscan Pro 6000 (distributed by Welch Allyn) [27]. More frequent observations were made for patients depending on clinical judgement and standard care protocols in place in the clinical areas. RR was recorded by the nurses through manual counting for one minute as per routine standard practice. This real-time observational data taken by the nurses acted as a comparison for the vital signs taken by the sensor.

A member of the research team collected the observation chart 24 h after the sensor had been placed. The observations for these times were recorded on an Excel sheet in a secure file held on the hospital server. If there was any uncertainty in the reading of the observation chart, this field was left blank and verified by a second reviewer. Vital signs were measured for all patients throughout their hospital stay. If a patient’s stay was longer than 5 days, a new sensor was placed.

### 2.8. Comparison of Sensor with Standard Practice

The sensor data were synchronised with manual observation times, creating paired datasets for comparison. Erroneous sensor data were flagged as invalid and excluded from analysis. Simulations tested the optimal averaging time window for comparing sensor and ward observations, applying filters of 6, 10, 12, 16, and 18 min. A previous study used a median filter of 15 min [21].

The results showed minimal differences in correlation between ward observations and the sensor across the time windows tested. The 10 min window was ultimately chosen as it provided the best balance between maintaining a small monitoring interval while minimising data loss. Smaller time windows, such as 6 min, had fewer matched pairs, leading to a reduction in the available data for analysis. While the 6 min window demonstrated strong correlation, the significant loss of matched data pairs made it less practical. Furthermore, given that not all ward staff can complete observations in under 10 min, including ranges below this threshold could introduce inconsistencies.

Conversely, larger averaging time windows, such as 16 or 18 min, posed a risk of missing important early physiological changes. Patients may enter a new physiological process during these longer intervals, potentially delaying the detection of deterioration. The 10 min window was therefore determined to be the most clinically appropriate, ensuring a reliable balance between timely detection and data integrity.

### 2.9. Statistical Analysis

A formal power calculation was not feasible due to a lack of preliminary data, but a sample size of 500 patients was chosen to ensure sufficient data on patient deterioration. The mean differences between nursing observations and sensor readings were calculated for each vital sign.

The one-sided T-test was performed. Agreement between two methods of clinical measurement was assessed with a Bland–Altman analysis of repeated measures [12,21,28]. To quantify the reliability and validity of sensor-derived measurements compared to the clinical (ward observations) reference standard, intraclass correlation coefficients (ICCs) were computed using a two-way random-effects model whenever sufficient repeated measures were available. In instances where specific time window analyses resulted in too few repeated data points to allow for reliable ICC estimation (e.g., very short averaging windows with limited matched pairs), Spearman’s correlation was used instead. Patients with only a single observation pair were excluded from ICC calculations where appropriate to preserve methodological integrity. Agreement levels for ICC values were defined as follows:

- 0.00–0.39: poor to fair agreement;
- 0.40–0.59: moderate agreement;
- 0.60–0.74: good agreement;
- 0.75–1.00: excellent agreement;

Additionally, Kappa agreement was analysed to assess the level of consistency between sensor readings and nurse observations. Histograms were created to visually inspect the distribution of values. Data were analysed using IBM SPSS Statistics (Version 25), R (version 4.20) using the “ggplot” and “MethComp” packages, and STATA/SE (Version 15.1).

## 3. Results

Between January 2018 and January 2019, a total of 1398 patients were screened, of whom 500 were recruited into this study. Depending on the length of hospital stay, sensor data were collected for an average of 2–3 days, extending longer for patients with prolonged admissions.

### 3.1. Reliability, Data Points, and Time Windows

A total of 27,397 ward data points were recorded for HR, RR, and temperature. The proportion of valid readings was high: 95.7% for HR, 97.2% for RR, and 95% for temperature. For nursing observations, the rates of illegible readings were 2.2% for HR, 2% for temperature, and 0.8% for RR, while missing data points accounted for 1.6%, 1.5%, and 2.4% of HR, RR, and temperature readings, respectively.

Determining the optimal averaging time window for comparing sensor data with ward observations is complex, with variations reported in the literature. Large time windows can result in lower correlation due to expected changes in vital signs over time, particularly for ambulatory patients. Conversely, short time windows are more vulnerable to outlier values and increased data loss due to clustered invalid readings caused by patient activities such as talking or eating.

Five averaging time windows (6, 10, 12, 16, and 18 min) were assessed (Table 1). The 10 min window provided the smallest monitoring interval with minimal data loss and sufficient paired observations. Shorter windows, such as 6 min, had significant data loss, while larger windows risked capturing physiological changes occurring within the interval. Therefore, the 10 min window was selected for analysis.

### 3.2. Agreement Between Sensor-Derived and Ward-Based Measurements

A 10 min averaging window was used to assess the agreement between sensor-derived and ward-based physiological measurements. The mean differences (Table 1) were 3.63 bpm (HR), −2.72 breaths per minute (RR), and −0.57 °C (T). The limits of agreement (LoA), representing the range within which 95% of the differences are expected to lie, were −10.87 to 18.14 bpm for heart rate, −10.91 to 5.47 breaths per minute for respiratory rate, and −1.72 to 0.58 °C for temperature. These findings indicate a moderate level of agreement between the two measurement methods, with HR showing the narrowest LoA, suggesting a more consistent relationship between the two modalities. By contrast, RR and T exhibited greater variability, which may reflect inherent challenges in respiratory rate monitoring and differences in temperature measurement sites.

**Figure 2:**
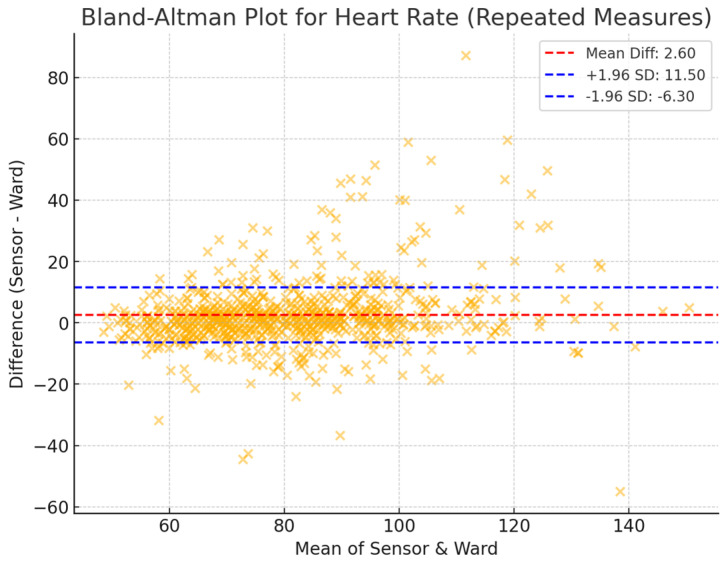
Bland–Altman plots of Sensium HR and ward observations in a 10 min averaging time window.

**Figure 3:**
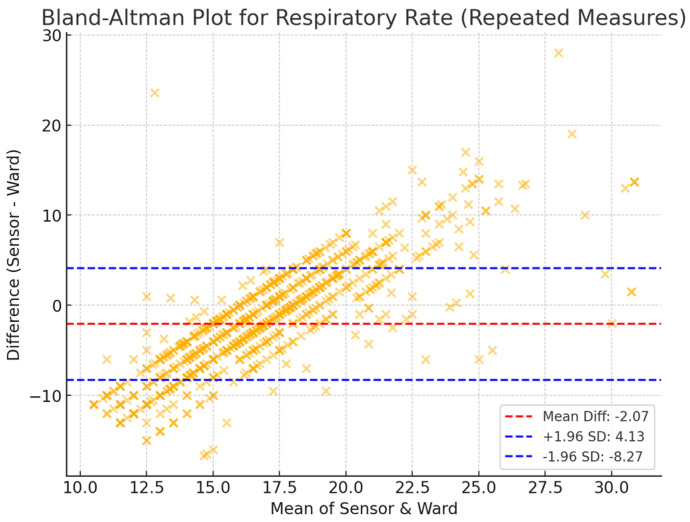
Bland–Altman plots of Sensium RR and ward observations in a 10 min averaging time window.

**Figure 4:**
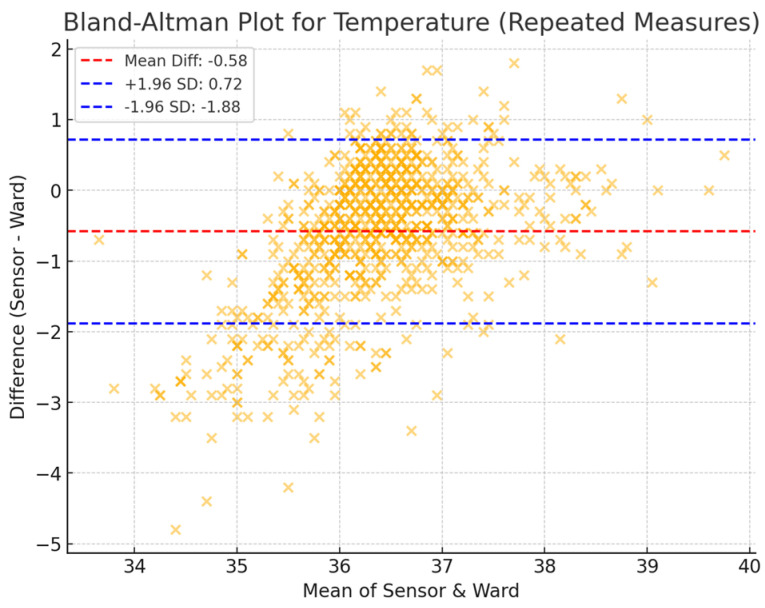
Bland–Altman plots of Sensium temperature and ward observations in a 10 min averaging time window.

The Bland–Altman plots of repeated measures (Figure 2, Figure 3 and Figure 4) further illustrate the distribution of measurement differences across the range of values. HR displayed consistent agreement with ward measurements, with no significant trend in bias over different heart rate ranges. In contrast, RR exhibited a wider scatter, with a greater degree of variability across measurements, highlighting the challenge of accurately capturing respiratory rate through sensor-based monitoring. Temperature readings showed systematic underestimation by the sensor compared to ward-based observations, particularly at higher temperatures, which may be attributed to anatomical differences in measurement locations.

### 3.3. Reliability and Validity of Sensor-Derived Measurements

Intraclass correlation coefficients (ICCs) were used to evaluate the reliability of sensor-derived measurements compared to ward-based readings. HR demonstrated good agreement (ICC = 0.66, *p* < 0.001), and T showed fair agreement (ICC = 0.30, *p* < 0.001), whereas RR exhibited poor reliability (ICC = 0.20, *p* < 0.001), suggesting higher variability in respiratory rate measurements. These results indicate that HR measurements obtained from the sensor are relatively consistent with ward-based readings, whereas RR and T exhibit greater variability, which may limit their clinical applicability.

**Figure 5:**
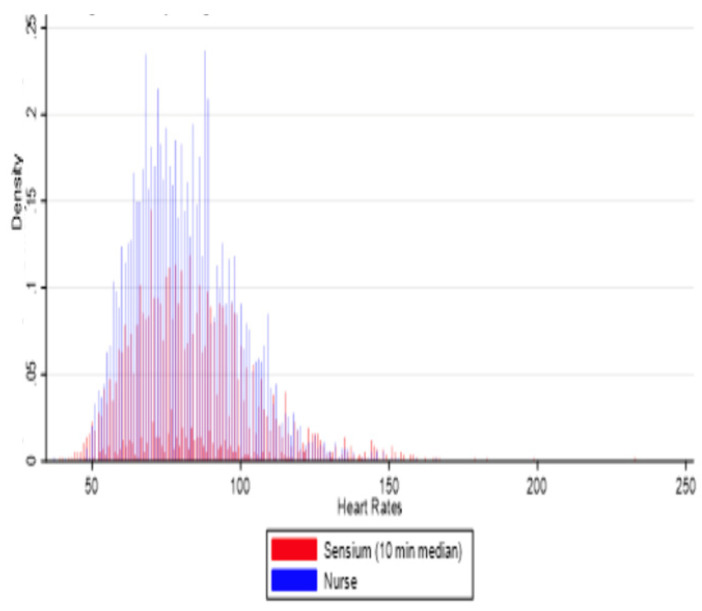
Histogram for the median matched pairs of Sensium HR and ward observations in a 10 min averaging time window.

For heart rate, a histogram (Figure 5) revealed that the sensor was able to make higher and lower readings overall. The average difference between the sensor and ward readings ranged from 2.4 to 2.9 bpm. The Kappa agreement between the nurses’ observations and sensor readings was 97%, indicating strong agreement.

**Figure 6:**
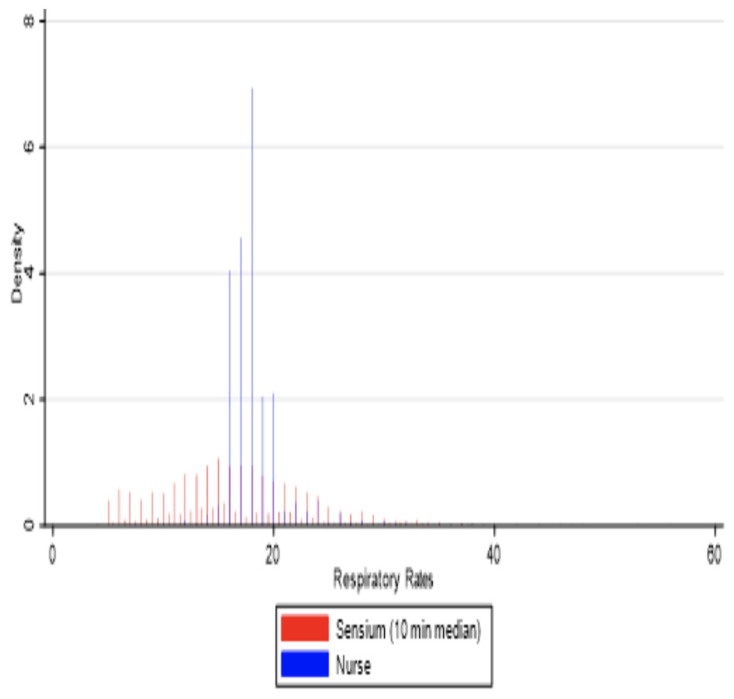
Histogram for the median matched pairs of Sensium RR and ward observations in a 10 min averaging time window.

For respiratory rate, a histogram (Figure 6) illustrated that nurse-recorded RR values were most commonly between 16 and 20 breaths per minute, with 18 being the most frequently observed. In contrast, sensor data exhibited a broader distribution, often reporting lower RR values compared to ward observations. The average difference between the two methods ranged from 1.7 to 1.8 breaths per minute, with an 82% Kappa agreement, reflecting moderate consistency between the sensor and manual measurements.

**Figure 7:**
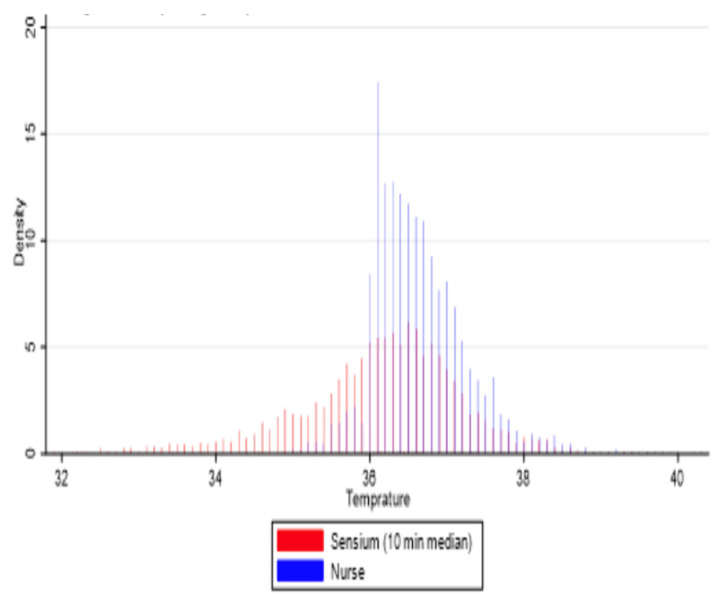
Histogram for the median matched pairs of Sensium temperature and ward observations in a 10 min averaging time window.

For temperature, a histogram (Figure 7) reveals that the temperature taken by the sensor axillary temperature was lower than the tympanic temperature taken by the nurses. The average difference between the sensor and ward observation readings ranged from −1.72 to 0.58 °C. The Kappa agreement between the nurses’ observations and the sensor for temperature was 60%.

## 4. Discussion

This study explored the real-world reliability of continuous monitoring for heart rate (HR), respiratory rate (RR), and temperature in acutely unwell hospital patients using a wearable sensor over multiple days. The results demonstrate that sensor-derived HR measurements exhibit strong reliability, with a mean difference of 3.63 bpm—substantially below the widely accepted threshold of 5 bpm [21,23,24,25,26,27,28,29,30] highlighting the sensor’s reliability for HR monitoring compared to standard ward observations, well within clinically acceptable limits. The narrow limits of agreement (LoA) further confirm the high level of consistency between the two modalities. By contrast, RR measurements exhibited greater variability, with a mean difference of −2.72 breaths per minute and poor reliability with other studies reporting an acceptable range of 2 breaths per minute [23,31], highlighting the inherent challenges of respiratory rate monitoring via impedance-based wearable sensors. However, this discrepancy may be attributable to inconsistencies in manual nursing observations, a well-documented limitation in clinical practice, where RR is often inaccurately recorded or subject to “data smoothing” [24,25,26,31,32,33,34,35], which biases observations toward expected normal values. Temperature readings showed fair agreement (ICC = 0.30), though a systematic underestimation was noted, particularly at higher temperatures.

One of this study’s key strengths is the detailed evaluation of averaging time windows for sensor data, addressing a significant gap in the literature. The 10 min window emerged as the most clinically appropriate, balancing data retention and real-time alerting. Shorter windows resulted in excessive data loss, while longer windows risked missing significant physiological changes. Additionally, median averaging proved more robust against outliers and skewed data, corroborating results from prior pilot studies [12].

A comparable study by Breteler et al. evaluated multiple wearable sensors for vital sign monitoring in high-risk surgical patients, using ICU-grade monitors as the reference standard [35]. Their findings also demonstrated strong agreement for HR across different sensor technologies, with the SensiumVitals sensor displaying a bias of −0.8 bpm and limits of agreement comparable to those observed in the present study. However, Breteler et al. reported broader limits of agreement for RR (−7.4 to 5.6 breaths per minute), similar to our findings, reinforcing the known challenges of RR monitoring in mobile patients. Additionally, Breteler’s study noted significant variability in data loss across sensors, with transmission loss ranging from 13% to 34%, a key limitation in real-world monitoring that our study also encountered. Together, these findings highlight the need for continued refinement of RR algorithms and strategies to mitigate missing data in wearable monitoring systems.

This study’s single-hospital setting may restrict the generalizability of its findings to other institutions with varying patient demographics, care protocols, and workflows. Differences in infrastructure and staff familiarity with technology could influence sensor performance and adoption, warranting validation in diverse healthcare environments.

Real-time respiratory rate (RR) monitoring remains a notable challenge due to the impact of patient activities—such as walking, talking, or laughing—on thoracic impedance measurements [12]. These activities likely contributed to the observed discrepancies in RR readings. Validation of the sensor against data from mechanically ventilated patients, where RR is measured with greater precision, could help mitigate this limitation and improve the robustness of future analyses. Although the recruitment of 500 patients yielded a substantial dataset, it is possible that data saturation was not fully achieved. As this was a feasibility pilot study, conducting formal sample size calculations was difficult in the absence of preliminary data. Future studies should aim to include larger sample sizes and implement power calculations to ensure adequate representation of physiological variability across populations. Additionally, the reliance on manual ward observations as the reference standard may have introduced bias, particularly for RR, where manual measurements are prone to inaccuracies.

Further studies should aim to validate wearable sensors in diverse patient populations and clinical settings, including multi-centre trials that encompass varying hospital infrastructures and patient demographics. Special emphasis should be placed on refining algorithms for respiratory rate (RR) measurement, as this vital sign demonstrated weaker correlation due to challenges inherent in both manual and sensor-based measurements. Exploring sensor performance in specific clinical scenarios, such as during sepsis or acute respiratory distress, would provide a more nuanced understanding of their efficacy. Additionally, the integration of machine learning to enhance sensor data interpretation, reduce noise, and improve the reliability of alert systems could represent a transformative advancement in this field.

An important consideration is that, in this study, nurses did not have access to the continuous sensor data or real-time alerts during patient care. While this design allowed us to assess the sensor’s accuracy independently, it may also have influenced the observed levels of agreement, particularly for respiratory rate and temperature, which exhibited greater variability. Access to continuous, objective data streams could have supported nurses in making more accurate and consistent observations. Moreover, real-time insights into patient trends could have enabled earlier identification of deterioration and more timely clinical interventions, potentially improving patient safety. Future research should explore the clinical impact of integrating continuous sensor data directly into nursing workflows, both in terms of improving data reliability and influencing patient outcomes. Clinicians would benefit from research exploring the practical implementation of wearable sensors in routine care. Studies assessing the impact of continuous monitoring on clinical workflows, nursing workload, and patient outcomes are critical for understanding how these technologies can be seamlessly integrated into practice. Investigating patient and caregiver perspectives on wearable sensors, including comfort, usability, and trust in digital monitoring, will ensure adoption aligns with the needs of end users. Future research should also explore how continuous monitoring can support early-warning systems and complement existing protocols, such as the National Early Warning Score (NEWS).

Policymakers should prioritise studies that assess the cost effectiveness of wearable sensor technologies, particularly in comparison to standard care. Evaluating long-term outcomes, such as reduced ICU admissions, shorter hospital stays, and improved morbidity and mortality rates, will be critical for building a strong economic case. Additionally, research into the scalability and infrastructure requirements for widespread adoption of wearable monitoring systems will guide policy decisions on funding and implementation. Policymakers must also consider developing standardised guidelines for the regulatory approval and clinical deployment of wearable sensors to ensure safety, reliability, and interoperability across healthcare systems.

Collaborative efforts among academics, clinicians, and policymakers are essential to address broader questions about the ethical use of wearable sensors, including data privacy, security, and equitable access. Future research should explore how wearable sensors can contribute to population health management, particularly in underserved or remote areas where real-time monitoring could bridge gaps in care delivery. Ultimately, fostering interdisciplinary research and cross-sector partnerships will accelerate the integration of wearable sensor technologies into patient care, enabling smarter, safer, and more efficient healthcare systems.

## 5. Conclusions

This prospective cohort study highlights the potential of wearable sensor technology to revolutionise patient monitoring in hospital wards. Continuous monitoring of vital signs using wearable sensors offers a transformative opportunity to enhance patient safety and care efficiency. By enabling smarter, safer ward environments, this technology paves the way for a paradigm shift in hospital-based patient care.

## Figures and Tables

**Table 1:** A comparison of the different averaging time windows for sensor readings with both means and medians.

Time Window (mins)	HR Mean Difference (LoA), bpm	RR Mean Difference (LoA), Breaths per Min	Temp Mean Difference (LoA), Degrees Celsius	HR Agreement	RR Agreement	Temp Agreement	HR Matched Pairs, *n*	RR Matched Pairs, *n*	Temp Matched Pairs, *n*
6	3.55 (+/− 5.11)	−2.03 (−14.95, 10.89)	−0.51 (−2.95, 1.92)	ICC = −0.041	ICC = 0.069	Spearman = 0.013, *p* = 0.701	960 (322 patients)	758 (288 patients)	877 (307 patients)
10	3.63 (−10.87, 18.14)	−2.72 (−10.91, 5.47)	−0.57 (−1.72, 0.58)	ICC = 0.66	ICC = 0.20	ICC = 0.30	1171 (341 patients)	1046 (330 patients)	1083 (335 patients)
12	3.20 (−35.86, 42.26)	−2.19 (−14.57, 10.18)	−0.56 (−2.75, 1.63)	ICC = 0.41	ICC = 0.14	ICC = 0.23	1172 (342 patients)	1083 (337 patients)	1095 (335 patients)
16	3.40 (−44.28, 51.07)	−2.01 (−14.54, 10.52)	−0.53 (−2.96, 1.90)	Spearman = 0.11	Spearman = 0.0006	Spearman = 0.028	1181 (336 patients)	1136 (338 patients)	1108 (335 patients)
18	3.40 (−46.02, 52.82)	−1.97 (−14.39, 10.44)	−0.50 (−2.88, 1.88)	ICC = 0.256	ICC = −0.021	ICC = 0.074	2265 (355 patients)	2163 (354 patients)	2156 (349 patients)

LoA: limits of agreement; HR: heart rate; RR: respiratory rate; ICC: intraclass correlation coefficient.

## Data Availability

The raw data supporting the conclusions of this article will be made available by the authors upon reasonable request.

## Abbreviations

The following abbreviations are used in this manuscript:

RR	Respiratory rate
HR	Heart rate
NICE	National Institute for Health and Clinical Excellence
ICU	Intensive care unit
